# Trying to understand who seeks open relationships: an evolutionary perspective

**DOI:** 10.3389/fpsyg.2026.1762047

**Published:** 2026-03-17

**Authors:** Vlad Burtaverde, Bogdan Oprea, Peter Karl Jonason, Serban Andrei Zanfirescu, Stefan Cosmin Ionescu

**Affiliations:** 1 Department of Psychology, University of Bucharest, Bucharest, Romania; 2 Psychology, University of Bucharest, Bucharest, Romania; 3 Psychology, Cardinal Stefan Wyszynski University, Warsaw, Poland; 4 Department of Psychology, University of Padova, Padova, Italy

**Keywords:** consensual non-monogamy, life history theory, mating strategies, personality, sociosexuality

## Abstract

Even if monogamy is the most desirable form of romantic relationship in the modern era, humans engage in an array of mating strategies. We aimed to understand consensual non-monogamy (CNM) from an adaptationist perspective by developing a measure of CNM preferences (Open Relationship Questionnaire; ORQ) and linking it to various relationships and life outcomes. Following construct operationalization and a literature search of CNM-related attitudes and behaviors, we generated 69 items as the initial item pool for the questionnaire. In Study 1 (*n* = 271), four factors emerged: *negative perceptions toward CNM*, *emotional insecurity in open relationships*, *preferences for CNM*, and *discriminatory views of CNM*, which are related to the Dark Triad traits, life history strategies, mate value, sociosexuality, childhood maltreatment, and romantic love. In Study 2 (*n* = 272), we confirmed the internal structure of the ORQ and related it to childhood economic status and sexual fantasies. This research offers new and interesting insights into a specific, less (mis)understood mating strategy.

## Introduction

Even if monogamy is the most desirable form of romantic relationship, humans adopt varied mating strategies (Buss, 2008). Some choose to remain single to invest in their status and secure a better mate later (Apostolou, 2017). Others avoid long-term relationships and prefer short-term ones. People also pursue relationship structures such as consensual non-monogamy (CNM; Moors et al., 2017), with an estimated worldwide prevalence of 4–5% (Alexander, 2019). CNM refers to a relationship where partners agree to have extradyadic sexual or romantic relationships (Conley et al., 2013), distinct from infidelity, which is nonconsensual nonmonogamy.

The aforementioned examples led Buss and Schmitt (1993) to argue that biologically, humans are pluralistic in their mating strategies. They developed the sexual strategies theory (SST), which states that humans have a repertoire of mating strategies that vary on a continuum from short-term to long-term mating orientation. Individuals oriented toward short-term mating frequently engage in casual sex, invest less in their romantic relationships, and have many sexual partners and experiences. Those oriented toward long-term mating are more emotionally invested in their romantic relationships (Buss, 2008).

Relying on SST, we assume that monogamy, operationalized as being in a committed relationship that includes the possibility of serial polygamy (divorce and entering into another relationship), is a lifestyle that reflects long-term mating. We argue that monogamy is a trade-off humans make to maximize reproductive success, and that it depends on specific ecological characteristics. First, mating strategies are explained by many characteristics, with sex being among the most important. Men are more oriented toward short-term mating than women are (Jonason et al., 2009), since having multiple offspring with different partners increases the likelihood that their genes will survive, given differences in gestation and primary caregiving (Schmitt et al., 2001).

The extent to which mating strategies are reproductively advantageous for individuals depends on the environmental characteristics in which they grow, which reflects their life history strategies (Figueredo et al., 2006). People who grow up in harsh and scarce environments develop fast life history strategies (FLHSs) and are usually oriented toward short-term mating, impulsive, callous, risk-taking, sensation-seeking, and aggressive, in contrast to those who grow up in stable environments, who are more self-regulated, planning, and delayed gratification (Burtăverde and Ene, 2021).

Life history theory (LHT) and the environmental security hypothesis (ESH; Pettijohn and Jungeberg, 2004) are two theoretical frameworks that can explain why monogamy is the most desirable form of romantic relationships. ESH assumes that individual mate preferences shift with the security of their environment (Reeve et al., 2017). The evidence supports this theoretical framework. In areas with a relatively high cost of living, women place greater importance on men’s resource-accounting ability and less on men’s emotional characteristics (Anderson and Klofstad, 2012). In harsh ancestral environments, where resources are scarce, monogamy is likely the most adaptive mating strategy because it involves investing effort in long-term relationships and being collectivistic (Hofstede, 2011). However, Life-history theory should not be understood as a dualistic, bipolar account of human nature. Instead, organisms following the path of developmental plasticity and conditional adaptation, which assume that there is a structured interplay between the organism and its environment, shaped by natural selection to increase the capacity of individuals to track both their internal condition and their external environments and, integrating this information, adjust the development of their phenotypes accordingly (Ellis et al., 2009). As such, even if in some species polygamy represents an adaptive mating strategy in certain environments (Fedorka and Mousseau, 2002), from a Life-history perspective, in the modern and developed world, selective forces have shaped a competitive but orderly and controllable environment that is perceived as stable, and that enacts future-oriented reproductive strategies in a more gender equality oriented world (Zhu and Chang, 2019).

This view is supported by the high prevalence of extended-family structures in premodern societies (Buss, 2015). Currently, monogamy is less preferred in societies with high standards of living, where people can more easily gather resources. For example, in Luxembourg, the third-largest economy in the EU, 88 out of 100 marriages end in divorce, whereas in Romania, among the weakest economies, only 23 out of 100 marriages end in divorce (Statista, 2020). We can see similar differences between countries that differ substantially in terms of GDI (Statista, 2024). It is well known that in less developed countries there is a high level of conventionality and traditionalism, which also implies the traditional family (Inglehart and Baker, 2000). However, these cultural features should be seen as adaptive mechanisms facilitated by selection (Boyd and Richerson, 1985). As such, even if at a first glance cultural norms might be responsible for these differences, we can infer that those norms are the expression of environmental conditions and life history strategies, such as in less developed countries selection have favored environments that are more risk-taking and characterized by dominance hierarchies, and gender inequality (Zhu and Chang, 2019), where traditionalism and conformity have been the most adaptive cultural features. In contrast, in more developed countries, selective forces have favored environments that are more competitive but orderly and controllable, characterized by gender equality, status, and prestige, where the most adaptive cultural feature was individualism.

We argue that higher living standards in modern, individualistic societies reduce selection pressures, fostering sexual exploration. CNM structures offer both long-term (e.g., parental investment) and short-term (e.g., increased reproductive success) advantages, minus many of the costs associated with infidelity. Thus, the emergence and spread of CNM reflect changes in environmental and cultural factors.

People in CNM value consent, honesty, and communication as central to their relationships and negotiate these values with their partners (Andersson, 2022). Zero-sum thinking about love – the perception that one person’s love gained is another’s love lost is associated with increased CNM devaluation ratings (Burleigh et al., 2017). Those involved in a CNM relationship consume more pornography (Rodrigues et al., 2021) and report higher sexual satisfaction than non-CNM individuals do (Conley et al., 2018). People with negative attitudes toward those involved in CNM report stronger purity and antiegalitarian beliefs, lower openness to experience, and a greater desire for moral approbation (Cunningham et al., 2022). Haupert et al. (2017a) reported that education, income, religious affiliation, political affiliation, and geographical region are not associated with CNM engagement, suggesting that those who engage in CNM are similar in socio-cultural characteristics to the rest of the population.

Individuals involved in CNM are more oriented toward FLHSs than monogamous individuals (Mogilski et al., 2021). Women involved in CNM have earlier pubertal development, and CNM people are at high risk-taking, have less aversion to germs, are high in sociosexuality, more interested in sex, and are lower in mate retention tactics (Mogilski et al., 2017, 2019, 2021). Drawing on these findings, the authors argue that the stigma toward CNM individuals represents an evolutionary mismatch and can be understood as a negative attitude of others towards CNM practitioners because their risky and promiscuous lifestyle threatens social cohesion and cooperation (Mogilski et al., 2020), as they promote extreme intragroup competition (e.g., violent mating competition, mate/child desertion, disease transmission).

Even as CNM research grows, most studies use comparative designs to compare CNM with non-CNM populations on various individual differences (Mogilski et al., 2017). Another line of research uses qualitative methods to gain insight into CNM (Griebling, 2012). Although the utility of these studies is undeniable, we believe that to fully understand the CNM phenomenon, conceptualizing CNM to clarify its nature and operationalizing it through a psychometric measure are necessary. Researchers will gain new insights into CNM by gathering knowledge from people who may not yet be engaged in CNM but have a strong preference for it. Likewise, important knowledge may be gathered from people with strong negative attitudes toward CNM. Such an endeavor was initiated by Cohen and Wilson (2017), who developed a brief measure of the CNM attitude scale (e.g., “you must be in a monogamous relationship to be in love”). However, this is an eight-item unidimensional scale. We believe that brief measures are useful, but they fail to capture enough variance of the construct under consideration (Diamantopoulos et al., 2012). Relying on the aforementioned literature, we strongly believe that CNM orientation is a multidimensional construct.

Consequently, we aimed to develop a measure of CNM preferences. The hypothesized structure of this measure was (1) negative attitudes toward CNM, (2) preference for CNM, and (3) emotional insecurity in CNM. We developed a total of 69 items that reflected the draft of the questionnaire. We chose these three dimensions after we reviewed the literature on CNM and identified that (1) some people have negative attitudes toward CNM. We operationalized this view in the first dimension: (1) Negative attitudes toward CNM (i.e., *open relationships are against human nature*); (2) Despite this negative stigma, approximately 5% of people engage in CNM (Haupert et al., 2017b), which we operationalized in the scale of Preference for CNM (i.e., *I think I would be satisfied in an open relationship*). (3) Those who engage in CNM have unrestricted sociosexuality and are low in avoidant attachment, which we operationalized based on the Emotional Insecurity Scale (e.g., *I would feel hurt in an open relationship*).

Our second objective is to test the measure’s convergent and criterion-related validity. We expect the instrument’s dimensions to be related to sociosexuality, sexual fantasies, FLHS, childhood maltreatment, childhood economic status, Dark Triad traits, mate value, intimacy, passion, commitment in romantic relationships, and sexual orientation. As FLHSs are related to CNM (Mogilski et al., 2020), we measured FLHSs and other proximal and distal indicators, such as the Dark Triad traits, childhood maltreatment, and childhood socioeconomic status (Jonason et al., 2009; Figueredo et al., 2006). We measured sociosexuality and sexual fantasies because high sociosexuality is related to CNM involvement (Mogilski et al., 2017). We measured the love dimensions (i.e., intimacy, passion, and commitment), as CNM was low in avoidant attachment (Mogilski et al., 2021). Sexual orientation was measured because gay individuals are more interested in CNM (Conley et al., 2013). Finally, we measured mate value because high mate value has previously been associated with ease of attracting mates and mating success (Shackelford and Buss, 1997). Therefore, those high in mate value may be more interested in CNM relationships because they can easily find another partner if they lose their current one.

In Study 1, we developed the item pool and performed a principal component analysis. We also tested the measure’s convergent validity and internal consistency. In Study 2, a confirmatory factor analysis (CFA) of the structure identified in Study 1 was conducted.

## Study 1: developing the open relationship questionnaire

### Development of the item pool

We developed an item pool for the hypothesized structure of the ORQ: (1) Negative attitudes toward CNM, (2) Preference for CNM, and (3) Emotional Insecurity in CNM, by relying on research that investigated attitudes toward CNM and identified behavioral anchors related to CNM attitudes and perceptions (Burleigh et al., 2017; Conley et al., 2013), such as: providing companionship, promoting trust, and preventing boredom. We tried to develop as many items as possible, and obtained 69 items.

### Method

#### Participants and procedure

We used G*Power to estimate the necessary sample size for significant effect sizes. The minimum required sample size for effect size (*r*) was 0.25, with alpha set at 0.95, and the statistical power set at 0.95 was 197 participants. In this research, we relied on 271 undergraduate psychology students (*M_age_* = 20.44, *SD* = 4.02, 82.3% women). Of all participants, 44% declared they were in a romantic relationship. All the measures were administered online via Google Forms. The consent of participants was implicitly obtained by adding in the survey tool the following statement: By proceeding, you are affirming that you consent to take part in this research.” The database used in this research is available at: https://osf.io/82fjk/overview.

#### Measures

##### Open relationship questionnaire

The 69 items were *a priori* operationalized in the following three dimensions: (1) Negative attitudes toward CNM (i.e., *open relationships are against human nature*), (2) Preference for CNM (i.e., *I think I would be satisfied in an open relationship*), and (3) Emotional insecurity (e.g., *I would feel hurt in an open relationship*).

We assessed the Dark Triad traits via the Short Dark Triad measure (Jones and Paulhus, 2014). This questionnaire consists of 27 items, with nine items for each of the Dark Triad traits: Machiavellianism (e.g., *I like to use clever manipulation to get my way*), narcissism (e.g., *I like to get acquainted with important people*), and psychopathy (e.g., *People who mess with me always regret it*). Each item was rated on a five-point Likert scale (1 = *strongly disagree*; 5 = *strongly agree*). All the items were averaged to create indices for Machiavellianism (Cronbach’s *α* = 0.78), narcissism (*α* = 0.72), and psychopathy (*α* = 0.69).

We assessed sociosexuality via the revised sociosexual orientation inventory (Penke and Asendorpf, 2008). The measure consists of nine items that measure three dimensions of sociosexual orientation: sociosexual behavior (*α* = 0.85), sociosexual attitude (*α* = 0.85), and sociosexual desire (*α* = 0.84). Each item is rated on a nine-point response scale: Sociosexual behaviors (1 = *0*; 9 = *20 or more*), sociosexual attitudes (1 = *strongly disagree*; 9 = *strongly agree*), and sociosexual desires (1 = *never*; 9 = *at least once a day*).

We measured life history strategies via the *Mini-K* (Figueredo et al., 2006). This is a brief measure of life history strategies that consists of 20 items (i.e., While growing up, I had a close and warm relationship with my biological mother) rated on a seven-point Likert scale (−3 = *disagree strongly*; 3 = *agree strongly*).

Mate value was measured with six items from Graham-Kevan and Archer (2009) that target the following mate value attributes: attractiveness, personality, education, intelligence, career prospects, and social status. All the participants were asked to compare themselves with their acquaintances, and rate themselves on each item (1 = *strongly disagree*; 5 = *strongly agree*). We averaged the items to obtain an index of mate value (*α* = 0.84).

We measured childhood trauma with the short form of the Childhood Trauma Questionnaire (Bernstein et al., 2003). It consists of 25 items, rated on a five-point Likert scale (1 = *strongly disagree*; 5 = *strongly agree*) grouped into five dimensions: emotional abuse (e.g., *Family said hurtful things*), physical abuse (e.g., *hit hard enough to leave bruises*), sexual abuse (e.g., *was touched sexually*), emotional neglect (e.g., *made to feel important*), and physical neglect (e.g., not enough to eat). All the items were averaged to create the index for childhood trauma (*α* = 0.91).

We assessed romantic love with the Triangular Love Questionnaire (Sternberg, 1997). The measure consists of 36 items rated on a five-point item scale (1 = *strongly disagree*; 5 = *strongly agree*) that reflects three dimensions: intimacy (12 items; i.e., *I have a warm and comfortable relationship with my partner*), passion (12 items; i.e., *I cannot imagine another person making me as happy as my partner does*), and commitment (12 items, i.e., *I will always feel a strong responsibility for my partner; α* = 0.98). The internal consistency for the entire scale was 0.98. The Romanian version of all measures was achieved by a standard translation-back-translation method by a researcher proficient in both languages.

### Results and discussion

An exploratory factor analysis (EFA) was conducted with 271 participants from Romania, revealing a six-factor model for 69 items that accounted for 56.31% of the total variance. The model was rotated via the varimax method with maximum likelihood estimation, and the fit was assessed via multiple indices. The Kaiser-Meyer-Olkin (KMO) test verified the sampling adequacy for the analysis (KMO = 0.90). Bartlett’s test of sphericity indicated that the correlation structure is adequate for factor analysis (*χ*^2^[2346] = 15924.04, *p* < 0.001). Traditionally, EFA results are evaluated on the basis of the Eigenvalues of the factors, the Scree plot, and the factor loadings. However, these traditional methods have several limitations, such as subjectivity in the interpretation of the Scree plot and uncertainty in the number of factors to retain on the basis of the eigenvalue following the guidelines for fit statistics in EFA (Finch, 2020), we computed the Tucker-Lewis index (TLI), root mean square error of approximation (RMSEA) and Bayesian information criterion (BIC). These indices are typically used in confirmatory factor analysis (CFA), but Finch (2020) suggests their use for a more robust assessment of EFA results. The findings revealed that the TLI was relatively low at 0.81, indicating the poor performance of the factor solution. The RMSEA was 0.06 (10% CI [0.06, 0.07]). Additionally, the BIC was calculated as −907.04, suggesting a poor fit. Furthermore, some items did not load on any of the six factors (see Table 1). However, we inspected the content of this model and called the six factors as follows: F1: Negative perceptions toward CNM – lack of love, F2; Negative perceptions toward CNM – lack of morality, F3: Preference for CNM, F4: Emotional insecurity in open relationships, F5: Discriminatory view of CNM, and F6: Negative perceptions from traditional views (Table 2).

**Table 1:** EFA factor loadings of the 69 items (Model 1).

Factor and the number of factors	Factor						Uniqueness
	1	2	3	4	5	6	
43. Open relationships are just a form of non-commitment.	0.79						0.24
42. Open relationships are just an excuse for lack of fidelity.	0.76						0.22
32. Open relationships cannot provide the emotional security you need.	0.74						0.28
41. I do not think you can truly love one person and be with someone else.	0.73						0.33
44. Open relationship does not exist, only people who do not really love each other.	0.70						0.28
49. Open relationships are just an excuse to cheat on your partner.	0.68						0.27
48. Open relationships cannot last too long.	0.66						0.38
21. My children would grow up healthier in a traditional relationship.	0.62						0.54
31. People who are in open relationships fool themselves.	0.61						0.36
18. Open relationships are unstable, a dyiadic relationship is better.	0.56						0.54
36. In an open relationship, I would feel like a third wheel.	0.55						0.27
65. It is possible to genuinely love someone and be in an open relationship.	−0.50						0.49
16. I believe that an open relationship cannot satisfy your emotional needs.	0.52						0.63
13. A traditional couple relationship gives me security.	0.51						0.64
60. If my partner propose an open relationship, I would immediately refuse.	0.51						0.38
39. Open relationships have no way of working.	0.50						0.37
40. I cannot be in an open relationship because I am too jealous	0.49						0.51
6. A traditional couple relationship is the basis for a normal and functional family.	0.46						0.62
61. If a friend were to be offered an open relationship, I would suggest not accepting it.	0.46						0.51
3. The idea of an open relationship disgusts me.	0.46						0.60
47. You cannot have a peaceful life in an open relationship.	0.45						0.46
19. Those who are in open relationships are people who take a lot of risks.							0.84
20. Those around me would be disappointed if I were in an open relationship							0.84
51. Open relationships are for godless people.		0.84				−0.36	0.16
52. Only unfaithful people enter into open relationships.		0.80				−0.39	0.20
56. Open relationships are for people that are sexually obsessed.		0.74					0.41
59. To be in an open relationship means being sinful.		0.74				−0.31	0.32
58. Priests should not solemnize couples in an open relationship.		0.73					0.40
46. People in open relationships lack morality.		0.70				0.35	0.25
63. Open relationships are against human nature.		0.70					0.46
53. Marriages between persons in an open relationship should not be allowed.		0.69					0.47
50. People in open relationships have mental health issues.		0.64					0.53
54. To accept an open relationship is to allow yourself to be deceived.		0.63					0.32
38. People who are in open relationships do not respect themselves.		0.62				0.34	0.31
57. I would not agree to be the godfather/godmother of a couple in an open relationship.		0.59					0.53
45. People in open relationships are hypocrites.		0.58				0.39	0.33
55. Accepting an open relationship means letting yourself be lied to.		0.55					0.37
37. I would not accept one of my children to be in an open relationship		0.55					0.54
7. A dyadic couple relationship is an indicator of personal development.		0.41					0.68
4. I have nothing against people who are in an open relationship.		−0.41					0.77
9. I understand those who want to be in an open relationship.		−0.41					0.58
30. People are born for open relationships.		0.41					0.61
67. I would like to be in an open relationship.			0.80				0.20
68. An open relationship would be like a fantasy come true			0.76				0.37
23. I think I would be more relaxed in an open relationship.			0.75				0.40
12. I think an open relationship can allow me to experiment more opportunities.			0.75				0.37
69. An open relationship would satisfy all my needs related to relationships with other people.			0.73				0.36
17. An open relationship can drive away monotony and boredom from the couple.			0.70				0.42
1. I think I would be satisfied in an open relationship.			0.67				0.40
2. I do not mind the idea of an open relationship.			0.66				0.45
64. Open relationships allow you a life with more new experiences.			0.57				0.61
66. Open relationships allow you to explore your sexuality without constraints.			0.55				0.54
14. A dyadic romantic relationship forces you to devote yourself totally to one person.			0.52				0.70
27. For me, an open relationship is a compromise that I would not accept.			−0.50				0.31
11. A traditional couple relationship restricts my freedom.			0.47				0.73
8. I do not see myself satisfied in a traditional couple relationship.			0.43				0.74
15. I think that in an open couple relationship there are fewer conflicts and fights.			0.42				0.82
10. Better to be alone forever than in an open relationship.			−0.41				0.58
5. I believe that people can be happy and fulfilled in an open relationship.			0.35				0.63
62. Only closed-minded people cannot accept that for some open relationships are the most suitable.			0.30				0.85
22. An open relationship means an easier breakup.							0.80
24. I would feel hurt in an open relationship.				0.80			0.12
25. I would feel betrayed in an open relationship.				0.79			0.07
26. I would feel humiliated in an open relationship.				0.58			0.31
29. I would feel like the second option in an open relationship.				0.58			0.28
28. I would feel used in an open relationship.				0.51			0.34
35. People in open relationships should not raise children.					0.86		0.04
34. People in open relationships should not adopt children.					0.84		0.09
33. People in open relationships should not have children.					0.77		0.19

**Table 2:** EFA factor loadings of the items retained after modification indices (Model 2).

Factor and the number of factors	Factor				Uniqueness
	1	2	3	4	
63. Open relationships are against human nature.	0.78				0.36
56. Open relationships are for people that are sexually obsessed.	0.77				0.39
38. People who are in open relationships do not respect themselves.	0.66				0.38
50. People in open relationships have mental health issues.	0.66				0.55
37. I would not accept one of my children to be in an open relationship	0.58				0.53
57. I would not agree to be the godfather/godmother of a couple in an open relationship.	0.58				0.57
30. People are born for open relationships.	0.54				0.52
32. Open relationships cannot provide the emotional security you need.		0.75			0.28
18. Open relationships are unstable, a dyiadic relationship is better.		0.66			0.50
16. I believe that an open relationship cannot satisfy your emotional needs.		0.63			0.55
40. I cannot be in an open relationship because I am too jealous.		0.59			0.58
60. If my partner propose an open relationship, I would immediately refuse.		0.55			0.43
3. The idea of an open relationship disgusts me.		0.53			0.59
47. You cannot have a peaceful life in an open relationship.		0.50			0.45
65. It is possible to genuinely love someone and be in an open relationship.		−0.48			0.61
19. Those who are in open relationships are people who take a lot of risks.					0.83
23. I think I would be more relaxed in an open relationship.			0.76		0.37
12. I think an open relationship can allow me to experiment more opportunities.			0.76		0.34
17. An open relationship can drive away monotony and boredom from the couple.			0.70		0.40
14. A dyadic romantic relationship forces you to devote yourself totally to one person.			0.60		0.62
11. A traditional couple relationship restricts my freedom.			0.56		0.65
8. I do not see myself satisfied in a traditional couple relationship.			0.47		0.72
15. I think that in an open couple relationship there are fewer conflicts and fights.			0.46		0.78
34. People in open relationships should not adopt children.				0.90	0.07
35. People in open relationships should not raise children.				0.90	0.05
33. People in open relationships should not have children.				0.81	0.18

M = mean; SD = standard deviation.

**Figure 1:**
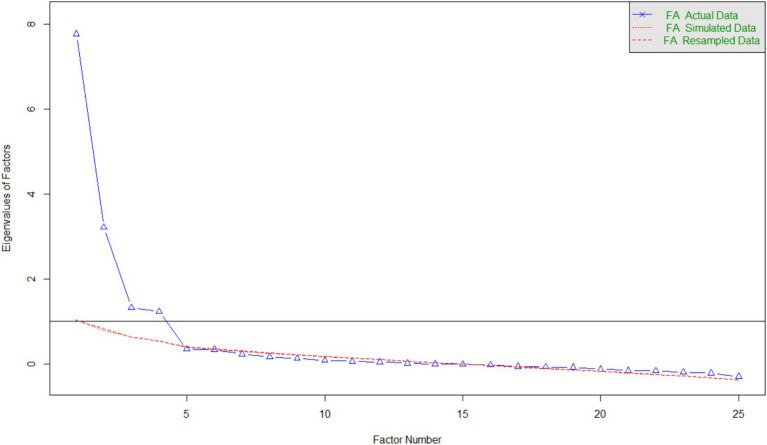
Screen plot regarding factor extraction - EFA (Study 1). FA = Exploratory Factor analysis.

After removing items on the basis of factor loadings, uniqueness, inter-item correlations, and theoretical application, 44 items were eliminated. EFA revealed a four-factor model that explained 54.12% of the data’s total variance. The varimax method with maximum likelihood estimation was used to rotate the factors. Bartlett’s test of sphericity indicated that the correlation structure was adequate (*χ*^2^[300] = 3894.19, *p* < 0.001), and the KMO score was 0.90, indicating that the data were suitable for factor analysis. The results showed that the four-factor model was a good fit for the data (*χ*^2^ = 323.68, *p* < 0.001; TLI = 0.95; RMSEA < 0.05 [0.04–0.06], BIC = −830.36). The factor loadings ranged from 0.46 to 0.90, and the scree plot (Figure 1) suggested that four factors were appropriate for this data set. Considering the content of the factors, we named them as follows: F1: Negative perceptions toward CNM (i.e., *Open relationships are for people who are sexually obsessed*), F2: Emotional insecurity in CNM (i.e., *Open relationships cannot provide the emotional security you need*), F3: Preference for CNM (i.e., *I think an open relationship can allow me to experiment more opportunities*), and F4: Discriminatory view toward CNM (i.e., *People in open relationships should not have children*). We can see that, to a great extent, the obtained internal structure of the ORQ reflected our *a priori* conceptualization, with the main difference being the presence of the fourth factor. Initially, the items of the fourth factor were conceptualized in the first factor – Negative perceptions of CNM.

Table 3 presents the mean and standard deviation for each factor of the ORQ based on sex. The sample included 48 males and 223 females. With respect to F1, F2, and F4, there were no differences between men and women. Men had higher scores on F3: Preference for CNM than women did.

**Table 3:** Descriptive statistics for the four factors.

Factor	Sex	*M*	*SD*	*t*-test	Effect size
F1: Negative perceptions towards CNM	Men	3.19	0.84	−1.06	−0.17
	Women	3.33	0.84		
F2: Emotional insecurity in CNM	Men	2.28	0.98	1.38	0.22
	Women	2.07	0.94		
F3: Preference for CNM	Men	2.57	0.85	2.48^*^	0.39
	Women	2.21	0.95		
F4: Discriminatory view towards CNM	Men	2.71	1.34	1.68	0.27
	Women	2.34	1.38		

^*^p < 0.05; *N* = 271 (*n*_Men_ = 48; *n*_Women_ = 223).

#### Convergent validity

The ORQ was related to the indicators used to test its convergent validity. Those that had high negative perceptions of CNM (F1) were high in mate value (*r* = 0.15, *p* < 0.05), passionate love (*r* = 0.12, *p* < 0.05), the commitment dimension of love (*r* = 0.12, *p* < 0.05), and low in sociosexuality (*r* = −0.30, *p* < 0.001).

Individuals high in emotional insecurity in the CNM (F2) reported high childhood maltreatment (*r* = 0.16, *p* < 0.01), passion (*r* = 0.18, *p* < 0.01), commitment (*r* = 0.20, *p* < 0.001), and low sociosexuality (*r* = −0.33, *p* < 0.001).

**Figure 2:**
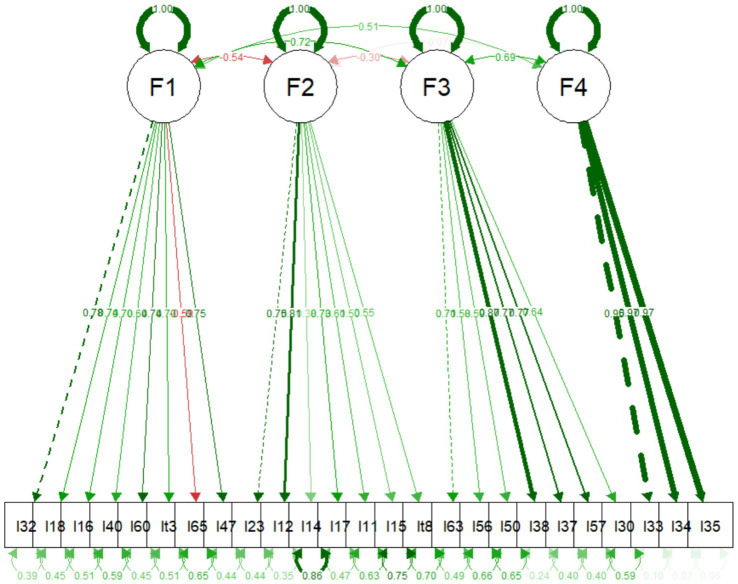
Factor loadings regarding the Confirmatory factor analysis.

Those who highly preferred CNM (F3) were high in sociosexuality (*r* = 0.35, *p* < 0.001), Machiavellianism (*r* = 0.28, *p* < 0.01), narcissism (*r* = 0.15, *p* < 0.05), psychopathy (*r* = 0.37, *p* < 0.01), and childhood maltreatment (*r* = 0.16, *p* < 0.01), and low in mate value (*r* = −0.14, *p* < 0.05), slow life history strategies (*r* = −0.15, *p* < 0.05), intimate (*r* = −0.14, *p* < 0.05), passion (*r* = −0.20, *p* < 0.01), and commitment (*r* = −0.23, *p* < 0.01), dimensions of love. Individuals who reported highly discriminatory of CNM (F4) had high mate value (*r* = 0.13, *p* < 0.05) (Table 4 and Figure 2).

**Table 4:** Correlation matrix (Study 1).

		1	2	3	4	5	6	7	8	9	10	11	12	13	14
1	F1: Negative perceptions towards CNM	—													
2	F2: Emotional insecurity in CNM	0.62^**^	—												
3	F3: Preference for CNM	−0.21^**^	−0.43^***^	—											
4	F4: Discriminatory view towards CNM	0.55^**^	.0.40^**^	0.01	—										
5	Socio Sexuality	−0.30^***^	−33.^**^	−35^**^	−0.10	—									
6	Mate Value	0.15*	0.08	−0.14^*^	0.13^*^	0.02	—								
7	Slow Life History	0.04	0.11	−0.15^*^	0.07	−0.07	0.57^***^	—							
8	Machiavellianism	0.11	0.04	0.28^**^	0.12	0.24^***^	0.07	−0.01	—						
9	Narcissism	0.05	0.01	0.15^*^	0.11	0.22^***^	0.48^***^	0.37^***^	0.36^***^	—					
10	Psychopathy	0.04	−0.11	0.37^**^	0.09	0.42^***^	−0.06	−0.18^**^	0.48^***^	0.32^***^	—				
11	Love Intimate	0.04	0.11	−0.14^*^	−0.03	−0.16^**^	0.25^***^	0.20^***^	−0.17^**^	0.13^*^	−0.14^*^	—			
12	Love Passion	0.12^*^	0.18^**^	−0.20^**^	−0.01	−0.21^***^	0.22^***^	0.18^**^	−0.14^*^	0.12	−0.14^*^	0.93^***^	—		
13	Love Commitment	0.12^**^	0.20^**^	−0.23	0.02	−0.24^***^	0.21^***^	0.18^**^	−0.17^**^	0.09	−0.14^*^	0.92^***^	0.95^***^	—	
14	Childhood Maltreatment	0.04	0.16^**^	0.16^**^	0.06	0.25^***^	−0.18^**^	−0.37^***^	0.13^*^	−0.09	0.31^***^	−0.06	−0.05	−0.06	—

*p < 0.05, **p < 0.01, ***p < 0.001.

## Study 2

### Participants and procedure

We used G*Power to estimate the necessary sample size for significant effect sizes. The minimum required sample size for effect size (*r*) was 0.25, with alpha set at 0.95, and the statistical power set at 0.95 was 197 participants. We relied on 272 undergraduate students in psychology (*M_age_* = 21.39, *SD* = 3.93, 83.8% women). Of all participants, 56% declared they were in a romantic relationship. The consent of participants was implicitly obtained by adding in the survey tool the following statement: By proceeding, you are affirming that you consent to take part in this research.” All the measures were administered online via Google Forms. The database used in this research is available at: https://osf.io/82fjk/overview.

### Measures

#### Open relationship questionnaire

We again administered the open relationship questionnaire.

#### Sexual fantasies

We assessed sexual fantasies with the Sexual Fantasies Questionnaire (Wilson, 1975), which consists of 40 items (10 items for each dimension) that encompass four dimensions: exploratory fantasies (i.e., *sex with two other people*), intimate fantasies (i.e., *having intercourse with a loved partner*), impersonal fantasies (i.e., *intercourse with an anonymous stranger*), and sadomasochistic fantasies (i.e., *being forced to do something*). The internal consistency for the entire scale was 0.93.

#### Childhood socioeconomic status

To measure childhood SES, we used established measures (Stamos et al., 2019). The participants were asked to indicate their agreement with three statements on a 5-point scale (1 = strongly disagree; 5 = strongly agree): “My family usually had enough money for things when I was growing up,” “I grew up in a relatively wealthy neighborhood,” and “I felt relatively wealthy compared to the other kids in my school.”

### Results

#### Confirmatory factor analysis

The CFA model, which was based on the four-factor structure found in the EFA, was applied to the second dataset (*n* = 272). The model yielded a significant chi-square test statistic (*χ*^2^ = 694.898, df = 293, *p* < 0.001), along with a comparative fit index (CFI) of 0.91 and a TLI of 0.90. The RMSEA was 0.07 (90% CI [0.06, 08]), indicating an acceptable fit to the data (Table 5). The standardized root mean residual (SRMR) was 0.07, which also suggests an adequate fit. Therefore, these results suggest that the CFA analysis produced a satisfactory model fit, and the factor loadings can be seen in Table 5. Next, we combined the samples from the two studies (*N* = 543) and loaded all the items onto a single factor. The purpose of the analysis was to assess the fit of the one-factor solution on the combined sample and compare it to the four-factor solution. The results indicated that the model did not fit the data well (CFI = 0.51, TLI = 0.49, RMSEA = 0.11 [0.11, 0.11] and SRMR = 0.12). These outcomes imply the presence of a multidimensional structure of the CNM (Table 6).

**Table 5:** CFA factor loadings of the items.

Factor	Indicator	*B*	*β*	SE
F2: Emotional insecurity in CNM	32. Open relationships cannot provide the emotional security you need.	1.09	0.78	0.07
	18. Open relationships are unstable, a dyiadic relationship is better.	0.98	0.75	0.07
	16. I believe that an open relationship cannot satisfy your emotional needs.	1.05	0.70	0.08
	40. I cannot be in an open relationship because I am too jealous.	0.79	0.64	0.07
	60. If my partner propose an open relationship, I would immediately refuse.	1.08	0.75	0.08
	3. The idea of an open relationship disgusts me.	1.10	0.70	0.09
	65. It is possible to genuinely love someone and be in an open relationship.	−0.78	−0.59	0.08
	47. You cannot have a peaceful life in an open relationship.	1.03	0.75	0.07
	19. Those who are in open relationships are people who take a lot of risks.	0.39	0.34	0.07
F3: Preference for CNM	23. I think I would be more relaxed in an open relationship.	0.80	0.75	0.06
	12. I think an open relationship can allow me to experiment more opportunities.	1.08	0.81	0.07
	14. A dyadic romantic relationship forces you to devote yourself totally to one person.	0.53	0.38	0.09
	17. An open relationship can drive away monotony and boredom from the couple.	0.90	0.73	0.07
	11. A traditional couple relationship restricts my freedom.	0.62	0.61	0.06
	15. I think that in an open couple relationship there are fewer conflicts and fights.	0.51	0.50	0.06
	8. I do not see myself satisfied in a traditional couple relationship.	0.53	0.55	0.06
F1: Negative perceptions towards CNM	63. Open relationships are against human nature.	0.79	0.71	0.06
	56. Open relationships are for people that are sexually obsessed.	0.57	0.58	0.06
	50. People in open relationships have mental health issues.	0.54	0.59	0.05
	38. People who are in open relationships do not respect themselves.	1.13	0.87	0.06
	37. I would not accept one of my children to be in an open relationship	0.94	0.77	0.06
	57. I would not agree to be the godfather/godmother of a couple in an open relationship.	1.16	0.77	0.08
	30. People are born for open relationships.	0.82	0.64	0.07
F4: Discriminatory view towards CNM	33. People in open relationships should not have children.	1.38	0.95	0.07
	34. People in open relationships should not adopt children.	1.41	0.97	0.06
	35. People in open relationships should not raise children.	1.41	0.97	0.06

**Table 6:** Correlation matrix (Study 2).

		1	2	3	4	5	6	7	8	9	10	11
1	F1: Negative perceptions toward CNM	—										
2	F2: Emotional insecurity in CNM	0.67^***^	—									
3	F3: Preference for CNM	−0.24^**^	−0.44^***^	—								
4	F4: Discriminatory view toward CNM	0.64^***^	0.48^***^	−0.06	—							
5	Exploratory fantasies	−0.27^***^	−0.33^**^	0.42^**^	−0.07	—						
6	Intimate fantasies	−0.24^**^	−0.10	0.20^**^	−0.05	0.56^***^	—					
7	Impersonal fantasies	−0.13^*^	−0.23^**^	0.41^**^	−0.05	0.77^***^	0.49^***^	—				
8	Sadomasochistic fantasies	−0.27^***^	−0.33^**^	0.40^**^	−0.08	0.72^***^	0.56^***^	0.63^***^	—			
9	Sexual fantasies	−0.27^***^	−0.30^*^	0.42^**^	−0.08	0.90^***^	0.78^***^	0.83^***^	0.87^***^	—		
10	Childhood socioeconomic status	0.15^**^	0.14*	−0.03	0.04	−0.02	0.01	−0.03	−0.02	−0.02	—	
11	Same-sex attraction	−0.21^**^	−0.30^**^	0.19^**^	−0.11	0.37^***^	0.15^*^	0.24^***^	0.34^***^	0.33^***^	−0.02	—

*p < 0.05, **p < 0.01, ***p < 0.001.

A multigroup confirmatory factor analysis was used to test for measurement invariance across men and women (Table 7). We began with a configural model, allowing the factor structure to be freely estimated in each group. We then tested for metric invariance, which constrains the factor loadings to be equal across groups. Finally, we tested for scalar invariance, which constrains the factor loadings and item intercepts to be equal across groups. The configural model demonstrated good fit with the data (CFI = 0.90, TLI = 0.89, RMSEA = 0.07, and SRMR = 0.07). The metric invariance model had a comparable fit, with only a slight increase in the SRMS value (0.08), indicating that the factor loadings were equivalent across groups. The scalar invariance model showed no differences in fit indices, suggesting that the item intercepts were equivalent across groups. These findings imply that the CNM measures the same construct with equal precision across males and females.

**Table 7:** Model fit comparison for measurement invariance.

	*χ* ^2^	CFI	TLI	RMSEA	SRMR	AIC	BIC
Configural fit	1354.86	0.90	0.89	0.07	0.07	38568.32	39264.45
Metric fit	1385.17	0.90	0.89	0.07	0.08	38556.63	39162.52
Scalar fit	1439.68	0.89	0.89	0.07	0.08	38569.14	39084.80

#### Convergent validity

Individuals with high levels of negative perceptions toward CNM (F1) reported high childhood socioeconomic status (*r* = 0.15, *p* < 0.01), few exploratory sexual fantasies (*r* = −0.27, *p* < 0.001), intimate sexual fantasies (*r* = −0.24, *p* < 0.01), impersonal sexual fantasies (*r* = −0.13, *p* < 0.05), sadomasochistic fantasies (*r* = −0.27, *p* < 0.001), general sexual fantasies (*r* = −0.27, *p* < 0.001), and low same-sex attraction (*r* = −0.21, *p* < 0.001).

Those with high emotional insecurity in the CNM (F2) reported high childhood socioeconomic status (*r* = 0.14, *p* < 0.05), and few exploratory sexual fantasies (*r* = −0.33, *p* < 0.001), impersonal sexual fantasies (*r* = −0.23, *p* < 0.001), sadomasochistic fantasies (*r* = −0.33, *p* < 0.001), general sexual fantasies (*r* = −0.30, *p* < 0.001), and low same-sex attraction (*r* = –0.30, *p* < 0.01).

Individuals who had a high preference for CNM (F3) reported many exploratory sexual fantasies (*r* = 0.42, *p* < 0.001), intimate sexual fantasies (*r* = 0.20, *p* < 0.001), impersonal sexual fantasies (*r* = 0.41, *p* < 0.001), sadomasochistic fantasies (*r* = 0.40, *p* < 0.001), general sexual fantasies (*r* = 0.42, *p* < 0.001), and high same-sex attraction (*r* = 0.19, *p* < 0.001).

### Discussion

The aim of this study was to develop a multidimensional scale to measure CNM. First, we investigated the scale’s internal structure and identified four factors. Three of them were initially proposed by us: negative perceptions toward CNM, emotional insecurity in CNM, and a preference for CNM. In addition to the three proposed factors, a new factor emerged, namely, discrimination toward the CNM. This finding is consistent with previous findings, which indicated that CNM is strongly stigmatized and perceived less favorably than monogamy is (Conley et al., 2013). Additionally, it converges with the evolutionary hypothesis according to which the stigmatization of sexual behavior outside the relationship is a deterrence mechanism against potential threats toward cooperation and group cohesion (Mogilski et al., 2020). This new instrument addresses the shortcomings of unidimensional scales of the CNM (e.g., Cohen and Wilson, 2017). The identification of the four components of the CNM allows research on specific antecedents (e.g., individual differences, social and economic factors) and consequences (e.g., jealousy, aggressive or sexual behaviors) for each type of attitude or preference related to the CNM.

Second, we tested the convergent and criterion-related validity of the ORQ. Individuals high in sociosexuality and those who reported having more sexual fantasies (such as exploratory, impersonal, and sadomasochistic) were less likely to have negative perceptions of CNM. These results are in accordance with studies that show positive links between involvement in open relationships and both sociosexuality (Mogilski et al., 2017) and pornography consumption (Rodrigues et al., 2021). Most likely, involvement in open relationships can represent, for people high in sociosexuality, a means to satisfy their sexual needs and fantasies. Therefore, these individuals will have positive perceptions of CNM.

As we expected on the basis of previous findings (Mogilski et al., 2020), negative perceptions of CNM were positively related to slow life history strategies and a distal antecedent of these strategies, namely, childhood socioeconomic status. Considering that a slow life history implies greater investment in parenting than in mating, slow life history strategists are more likely to prefer a closed relationship, which does not involve investing resources in other intimate relationships. Furthermore, the passionate love and commitment dimensions of love were positively related to negative perceptions of CNM. Consistent with studies indicating that people attracted to the same sex are more interested in open relationships (Conley et al., 2013), we found a negative correlation between same-sex attraction and negative perceptions of CNM.

Individuals who reported that they would be emotionally insecure in CNM scored higher on all dimensions of love and lower on sociosexuality and sexual fantasies. These relationships make sense from an evolutionary perspective because highly sociosexual people are more interested in sex without commitment and less concerned about emotional needs in intimate relationships. Therefore, they will not feel insecure in open relationships, but rather they will perceive CNM as an opportunity to enjoy short-term mating and various sexual experiences outside the main relationship. Moreover, emotional insecurity in CNM was negatively related to same-sex attraction. These results are convergent with the literature (Mogilski et al., 2017, 2021).

Consistent with previous findings (Mogilski et al., 2017), pro-CNM preferences were positively associated with sociosexuality. Additionally, people who reported more sexual fantasies had a greater preference for CNM, a result that is consistent with studies that show a positive link between pornography consumption and interest in CNM (Rodrigues et al., 2021). Their desire for casual sex can explain why highly sociosexual people prefer open relationships without commitment or closeness. Given the explicit consent of the partners to have sex with other people, they can satisfy these desires and live out their sexual fantasies.

Preference for CNM was positively related to FLHS and all the dark traits (Machiavellianism, narcissism, and psychopathy), and childhood maltreatment. As FLHSs imply an orientation toward sexual variety, little parental investment, and high mating investment, fast life history strategists will prefer open relationships because they can invest more in sexual opportunities outside the relationship. Previous studies support this explanation (Mogilski et al., 2020). The positive link between same-sex attraction and preference for CNM converges with previous findings (Conley et al., 2013).

Another indicator of the scale’s validity is that men reported higher preferences for CNM. The scale can highlight men’s stronger preference for short-term mating (Jonason et al., 2009) through access to partners outside the relationship. This difference between men and women can be evolutionarily explained by the greater benefits that men have as a result of this sexual strategy (Geary, 2016; Schmitt et al., 2001). We found no differences between men and women in terms of negative perceptions toward CNM, emotional insecurity in CNM, or discrimination toward CNM. Taken together, these results support the instrument’s convergent and criterion-related validity.

The present study has certain limitations. First, the samples consisted predominantly of women. Since there are important differences between men and women in terms of sexual behavior and preferences, the results could be distorted. Future studies should repeat the analysis on more balanced samples. Also, older participants might show greater tolerance of CNM. Thus, one cannot rule out the possibility that the findings are constrained by specific cultural backgrounds (e.g., samples from more individualistic societies) and the age group of the participants. The second limitation of the study is that we did not investigate the scale’s measurement invariance in relation to gender. Although we have identified differences between men and women in terms of preference for CNM, these differences could be due to the lack of equivalence for gender in measurement and not due to real differences between the preferences of men and women. Third, we did not differentiate between the types of open relationships (e.g., swings, group marriage, and polyfamilies). Future research should aim to identify what sets people who seek consensual open relationships apart from those who seek nonconsensual, extra-pair relationships (e.g., engage in infidelity). Moreover, ORQ represents an attitude oriented measure and might not reflect the true behavior of the participants in actual relationships.

Future research could use the ORQ to analyze attitudes and preferences for different types of open relationships. Finally, data were collected at the individual level. Future research based on the actor–partner interdependence model could include couples to investigate the role of preferences and attitudes toward open relationships in the dynamics between partners. To conclude, in this research, we offered some insights on who seeks consensual non-monogamous relationships by developing a measure of it and identified important individual differences that describe the psychological features of people interested in CNM.

## Data Availability

The datasets presented in this study can be found in online repositories. The database used in this research is available here: https://osf.io/82fjk/files/osfstorage?view_only=89ce804dc5e44cb38b942f8dc2b1d1ef.
